# Supplementary material for: Prospective Study on the Excretion of Mucous Stools and its Association with Age, Gender, and Feces Output in Captive Giant Pandas
Source: Animals (Basel). 2019 May 22;9(5):264. doi: 10.3390/ani9050264 (PMC6562534; doi:10.3390/ani9050264)
Supplement: Supplementary file 1 [file animals-09-00264-s001.pdf]

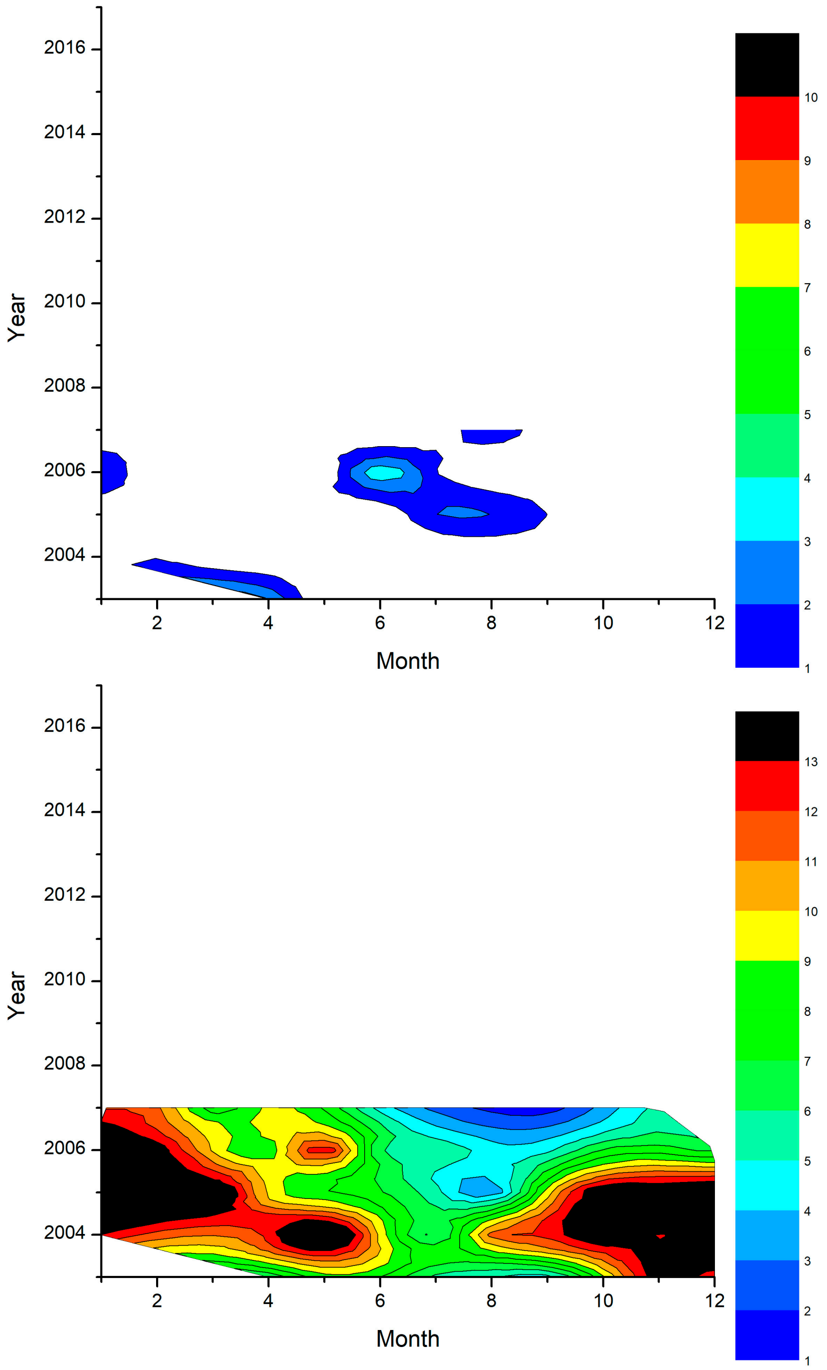
Year
2016
2014
2012
2010
2008
2006
2004
Month
2
4
6
8
10
12
10
9
8
7
6
5
4
3
2
1
Year
Month
13
12
11

(a)

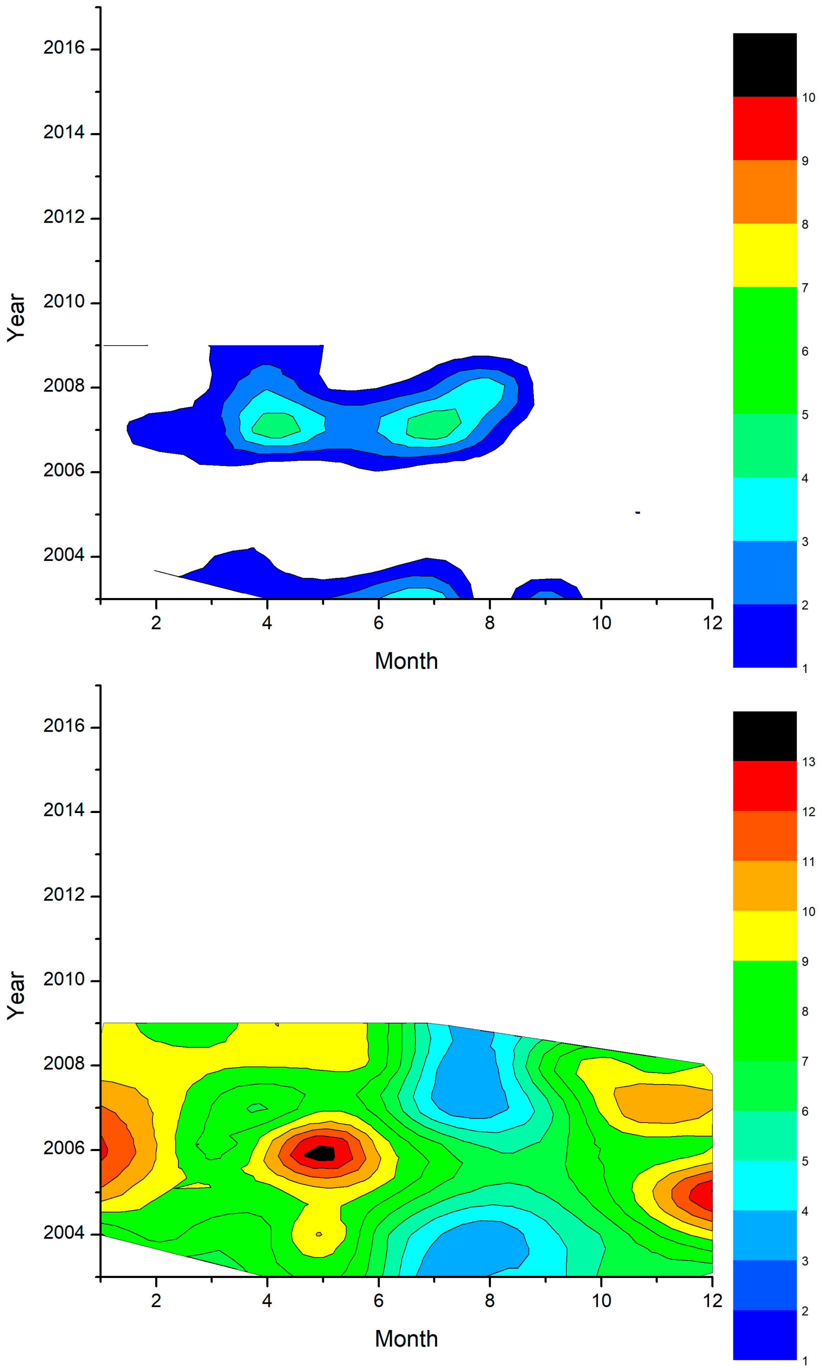
2016
2014
2012
2010
2008
2006
2004
Year
2
4
6
8
10
12
Month
10
9
8
7
6
5
4
3
2
1
2016
2014
2012
2010
2008
2006
2004
Year
2
4
6
8
10
12
Month
13
12
11
10
9
8
7
6
5
4
3
2
1

(b)

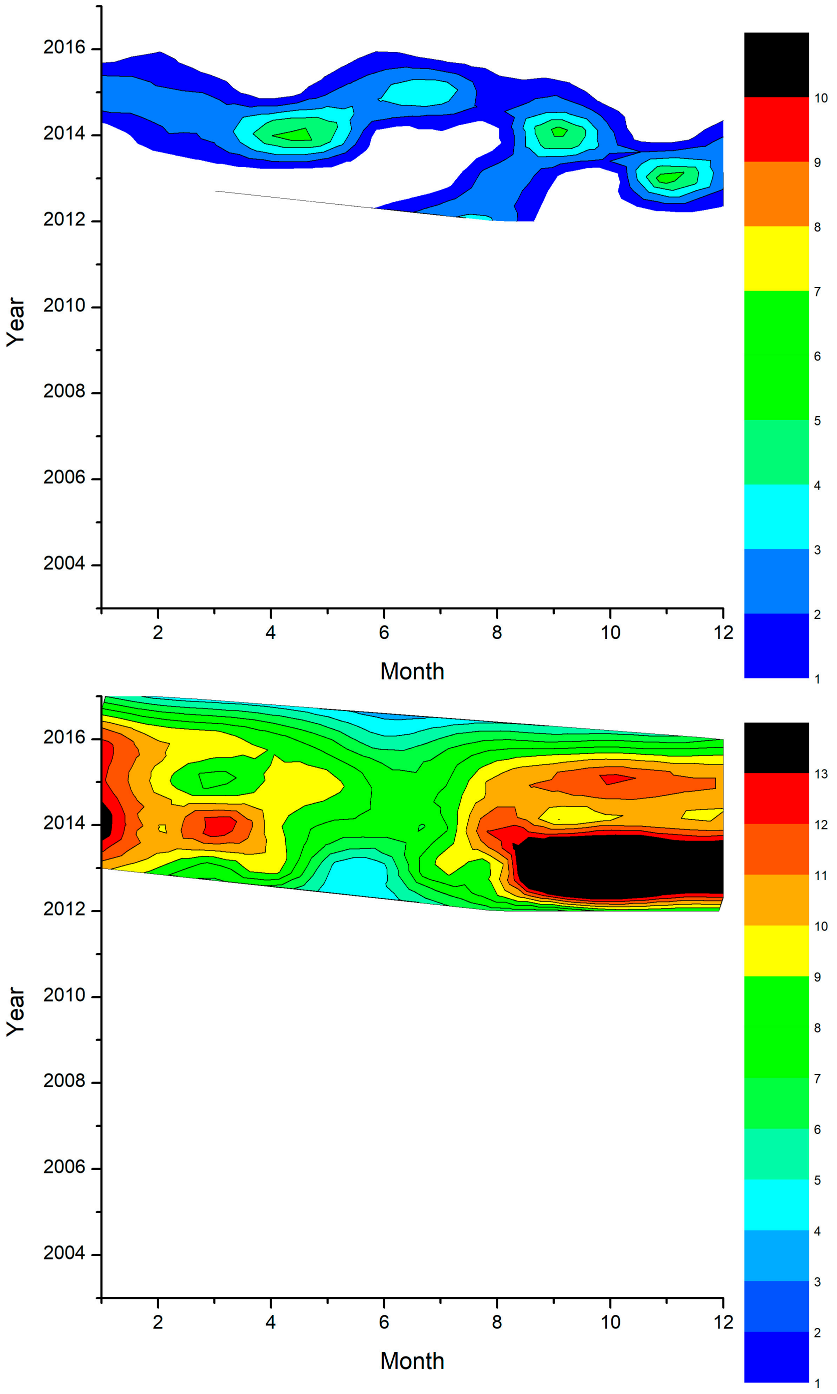
2016
2014
2012
2010
2008
2006
2004
Year
2
4
6
8
10
12
Month
10
9
8
7
6
5
4
3
2
1
2016
2014
2012
2010
2008
2006
2004
Year
2
4
6
8
10
12
Month
13
12
11
10
9
8
7
6
5
4
3
2
1

(c)

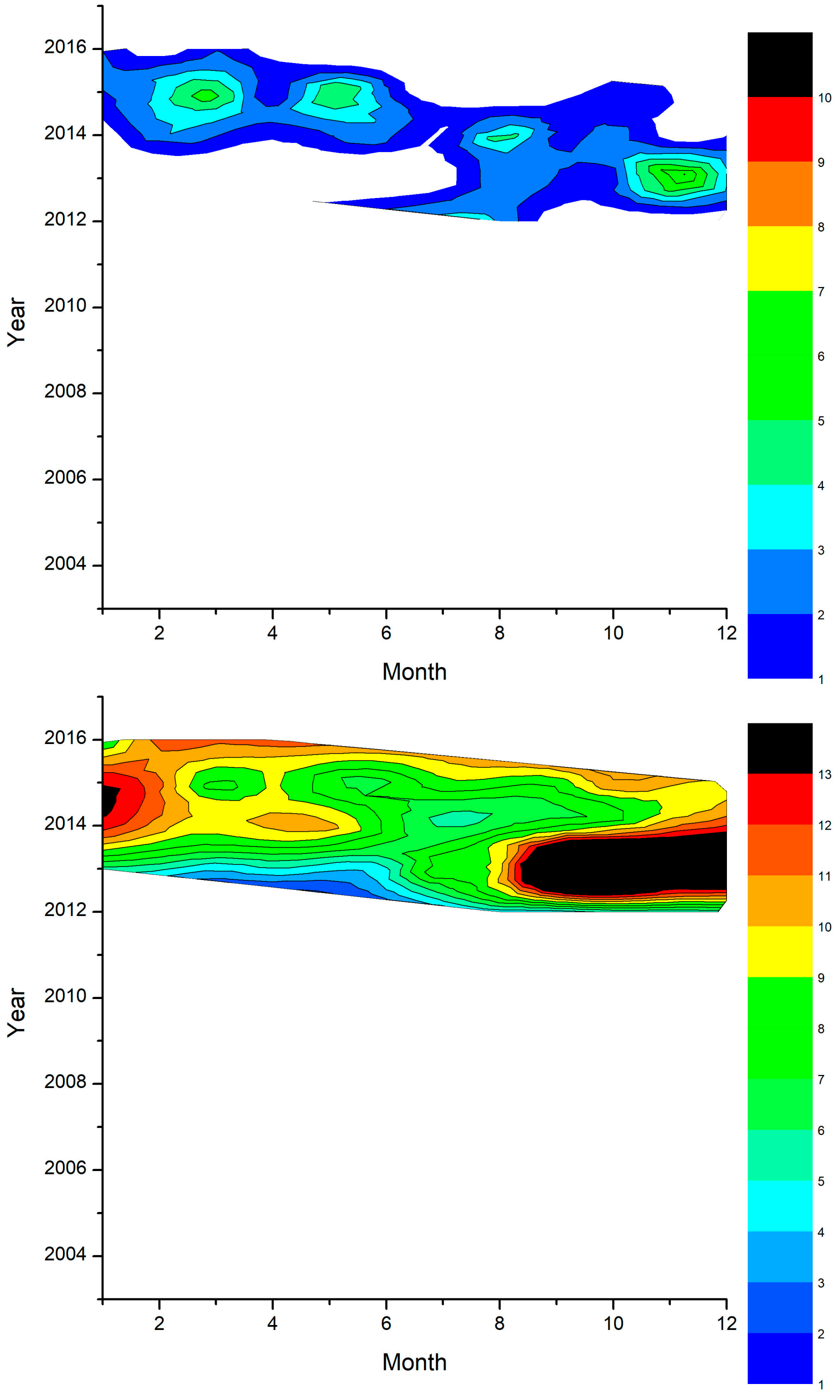
2016
2014
2012
2010
2008
2006
2004
Year
2
4
6
8
10
12
Month
10
9
8
7
6
5
4
3
2
1
2016
2014
2012
2010
2008
2006
2004
Year
2
4
6
8
10
12
Month
13
12
11
10
9
8
7
6
5
4
3
2
1

(d)

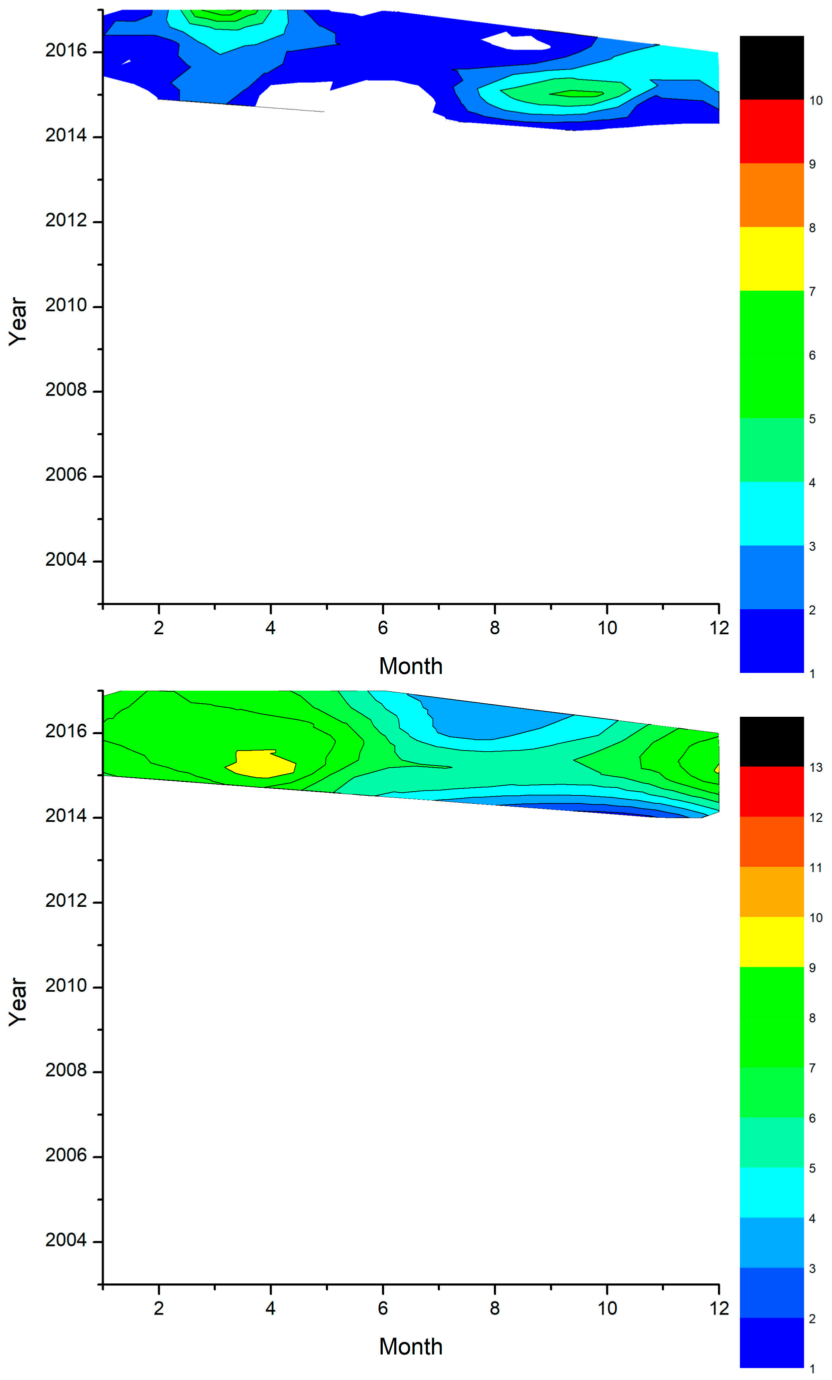

2016
2014
2012
2010
2008
2006
2004
Year
2
4
6
8
10
12
Month
10
9
8
7
6
5
4
3
2
1
2016
2014
2012
2010
2008
2006
2004
Year
2
4
6
8
10
12
Month
13
12
11
10
9
8
7
6
5
4
3
2
1

(e)

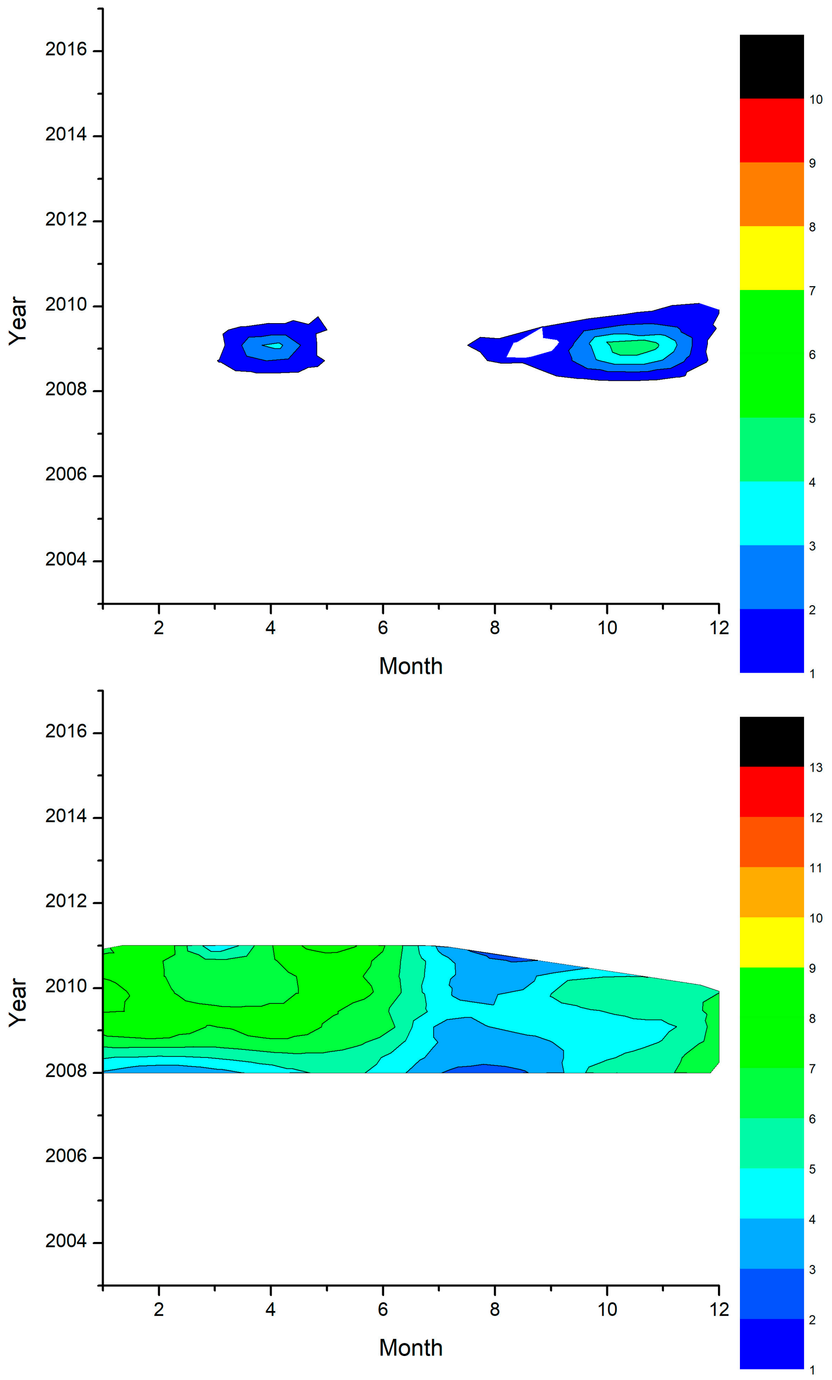
2016
2014
2012
2010
2008
2006
2004
Year
2
4
6
8
10
12
Month
10
9
8
7
6
5
4
3
2
1
2016
2014
2012
2010
2008
2006
2004
Year
2
4
6
8
10
12
Month
13
12
11
10
9
8
7
6
5
4
3
2
1

(f)

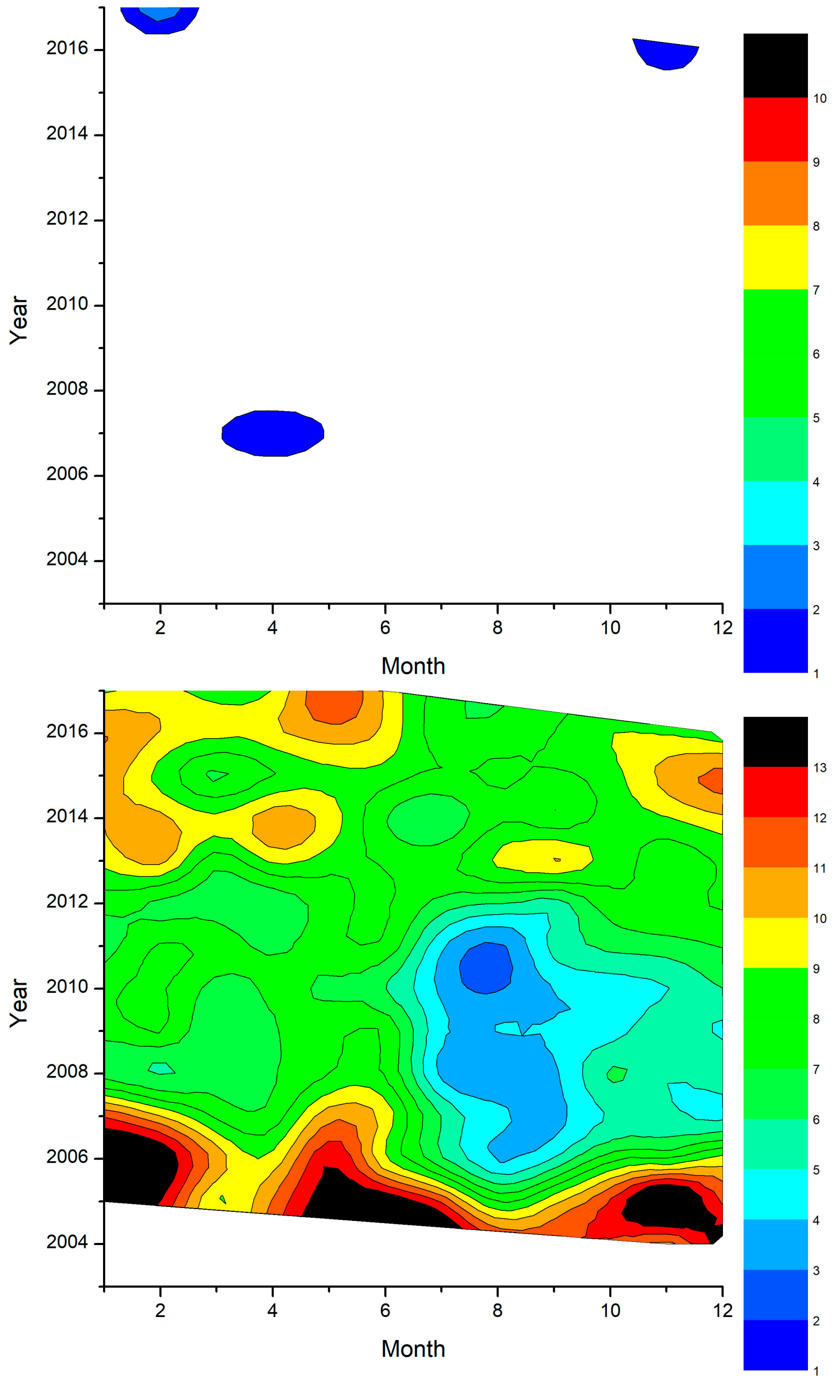

Year
2016
2014
2012
2010
2008
2006
2004
2
4
6
8
10
12
Month
1
2
3
4
5
6
7
8
9
10
11
12
13

(g)

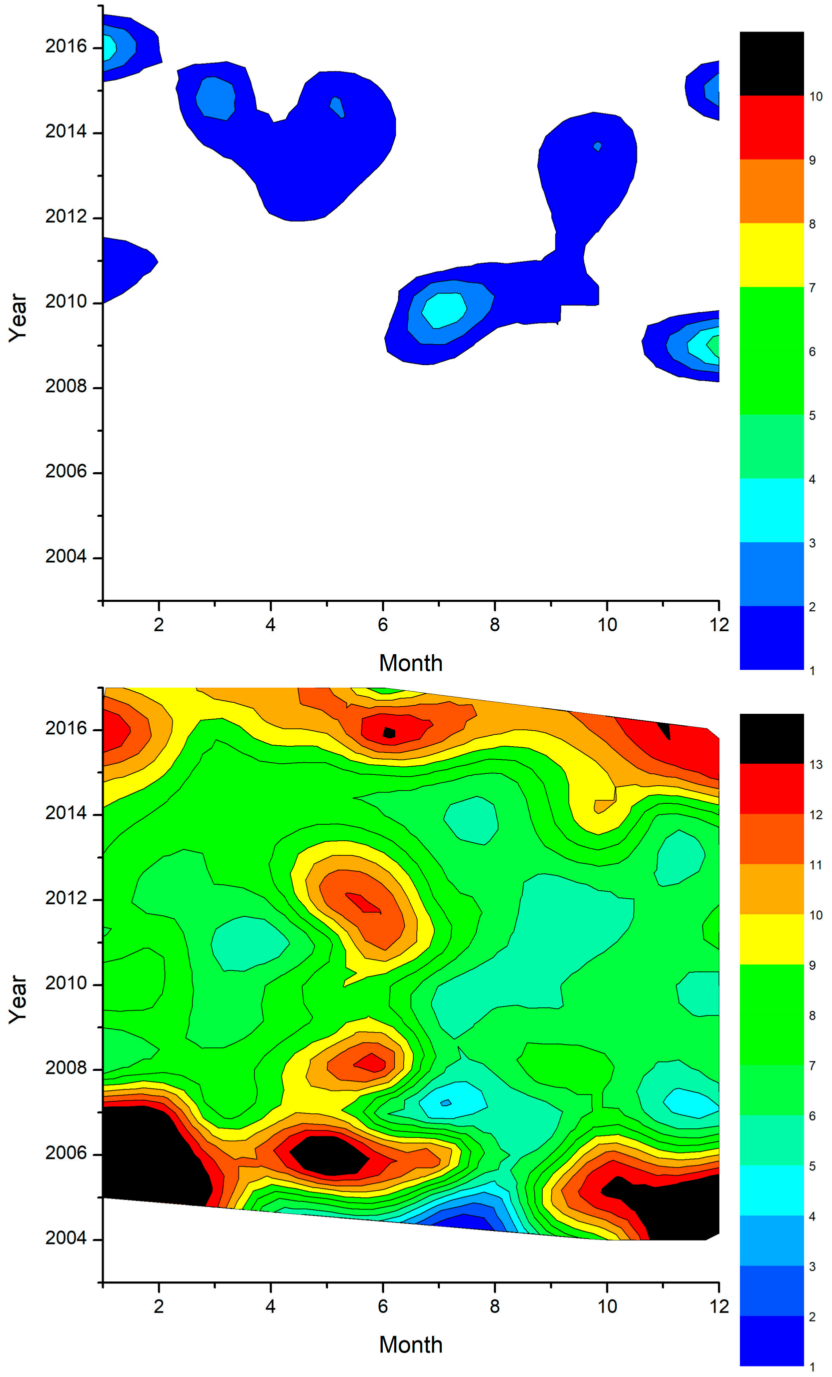
Year
2016
2014
2012
2010
2008
2006
2004
Month
2
4
6
8
10
12
1
2
3
4
5
6
7
8
9
10
11
12
13

(h)

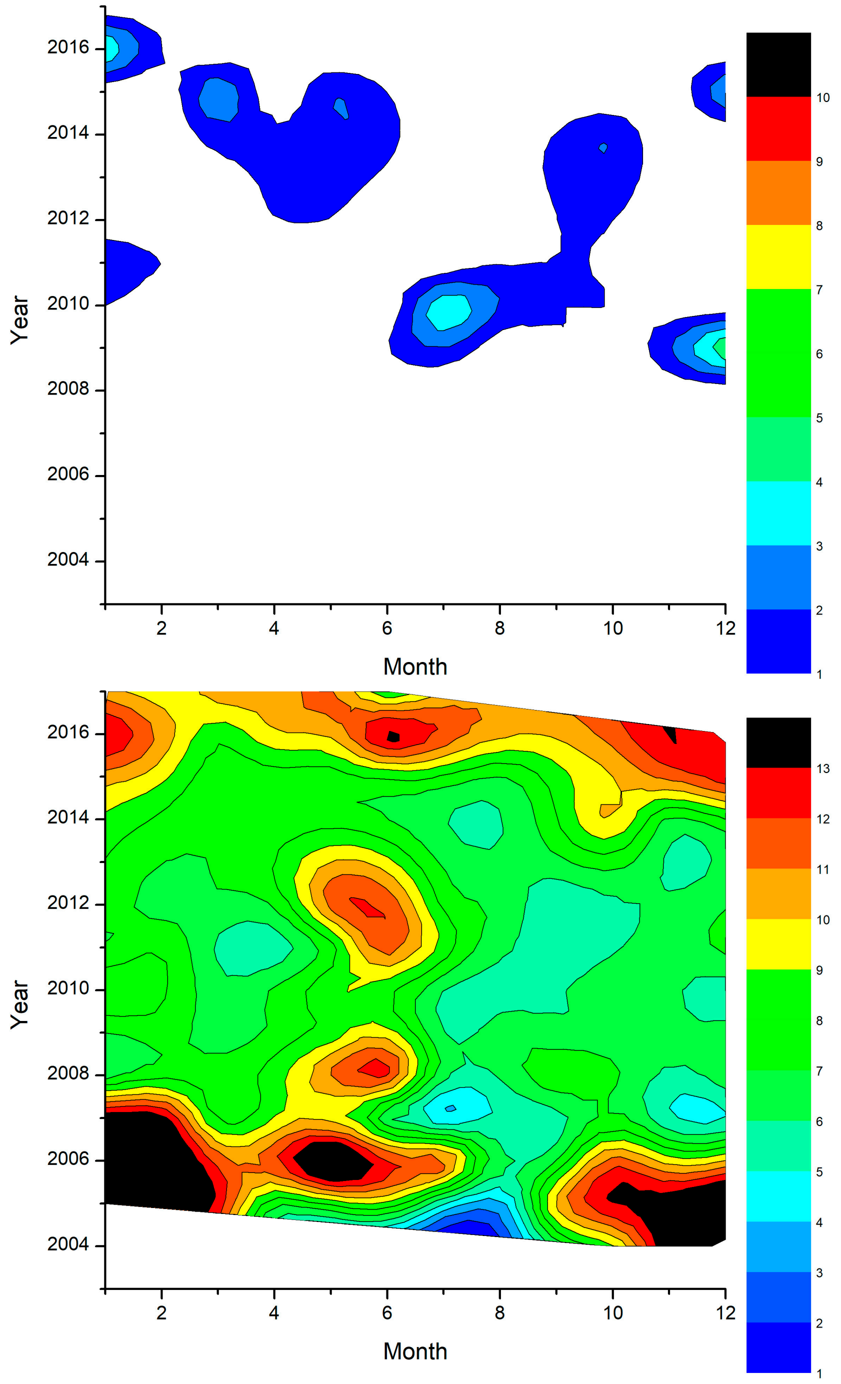
Year
Month
2016
2014
2012
2010
2008
2006
2004
2
4
6
8
10
12
1
2
3
4
5
6
7
8
9
10
Year
Month
2016
2014
2012
2010
2008
2006
2004
2
4
6
8
10
12
1
2
3
4
5
6
7
8
9
10
11
12
13

(i)

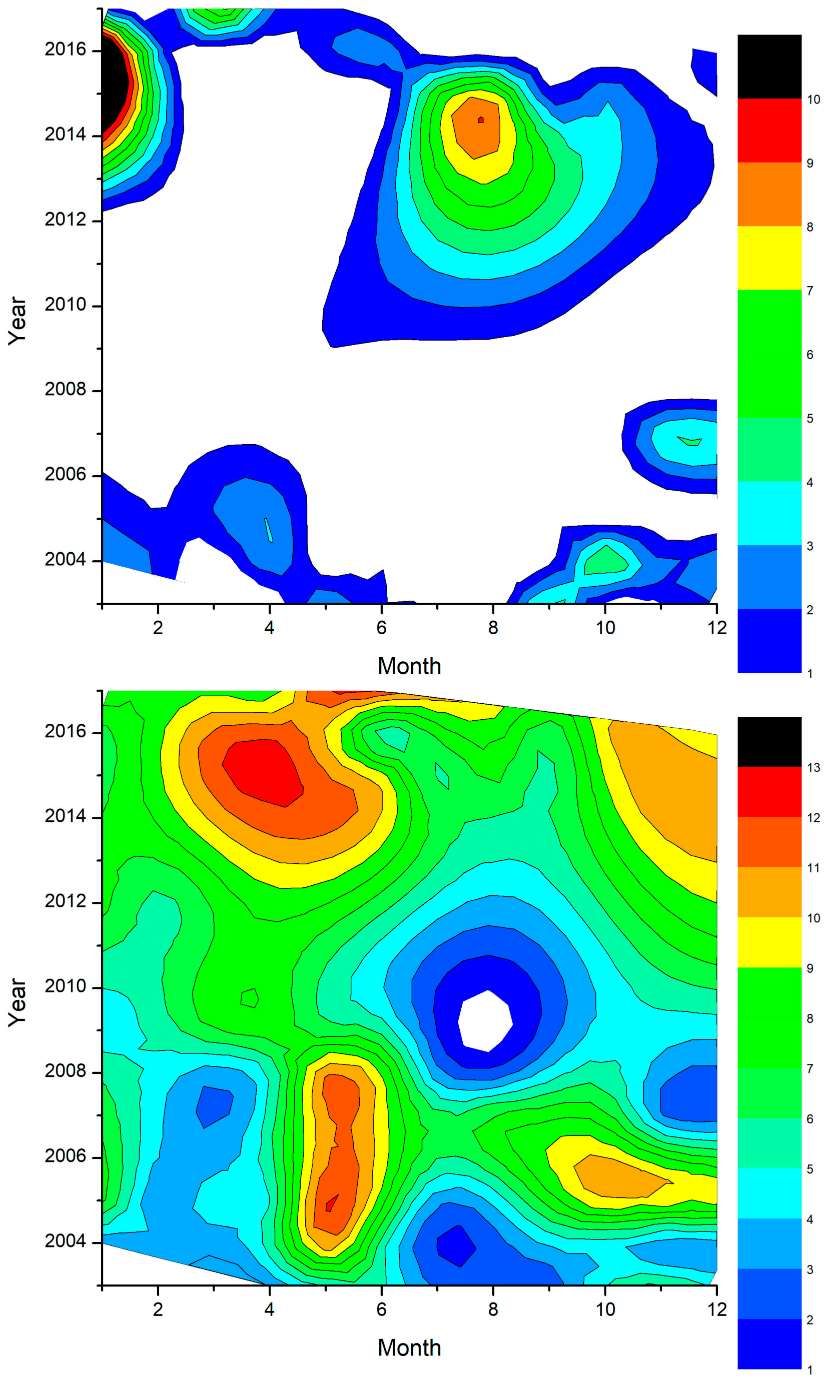

Year
2016
2014
2012
2010
2008
2006
2004
Month
2
4
6
8
10
12
1
2
3
4
5
6
7
8
9
10
Year
2016
2014
2012
2010
2008
2006
2004
Month
2
4
6
8
10
12
1
2
3
4
5
6
7
8
9
10
11
12
13

(j)

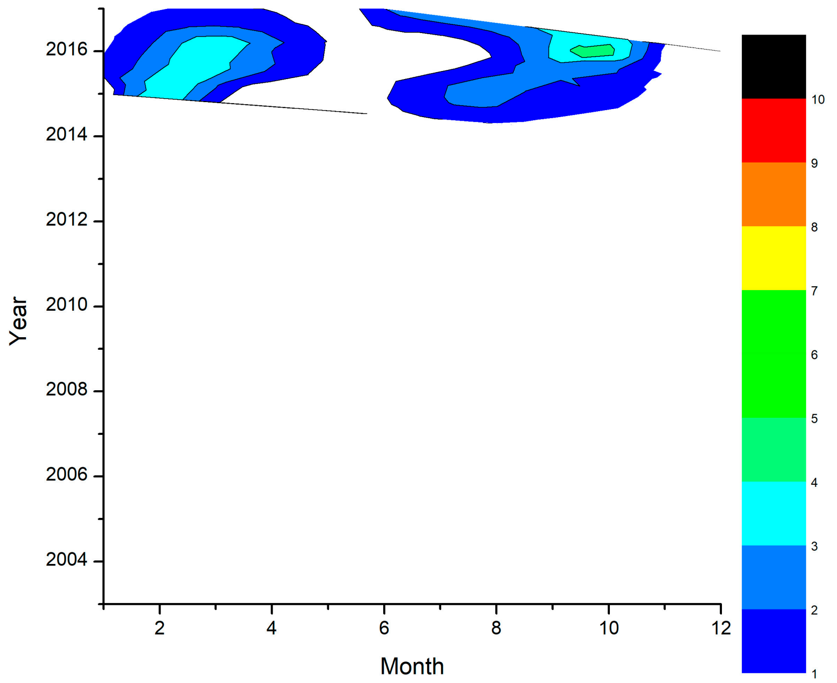

2016
2014
2012
2010
2008
2006
2004
Year
2
4
6
8
10
12
Month
10
9
8
7
6
5
4
3
2
1

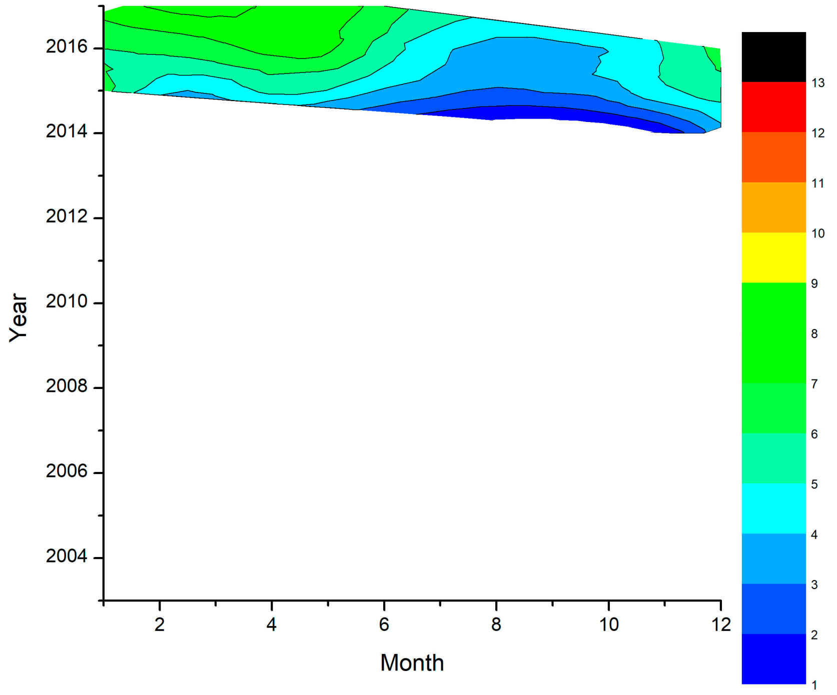

2016
2014
2012
2010
2008
2006
2004
Year
2
4
6
8
10
12
Month
13
12
11
10
9
8
7
6
5
4
3
2
1

(k)

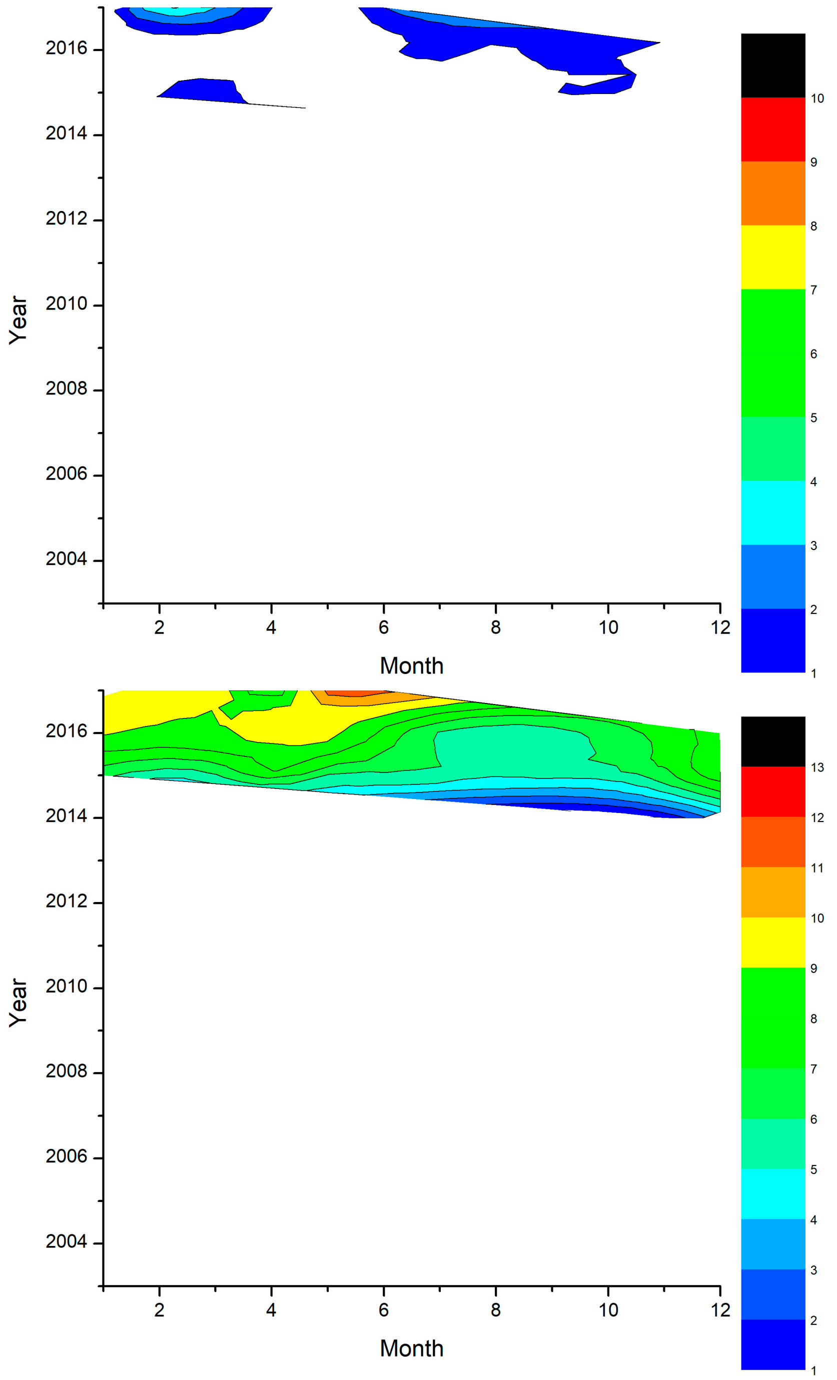

2016
2014
2012
2010
2008
2006
2004
Year
2
4
6
8
10
12
Month
10
9
8
7
6
5
4
3
2
1
2016
2014
2012
2010
2008
2006
2004
Year
2
4
6
8
10
12
Month
13
12
11
10
9
8
7
6
5
4
3
2
1

(l)

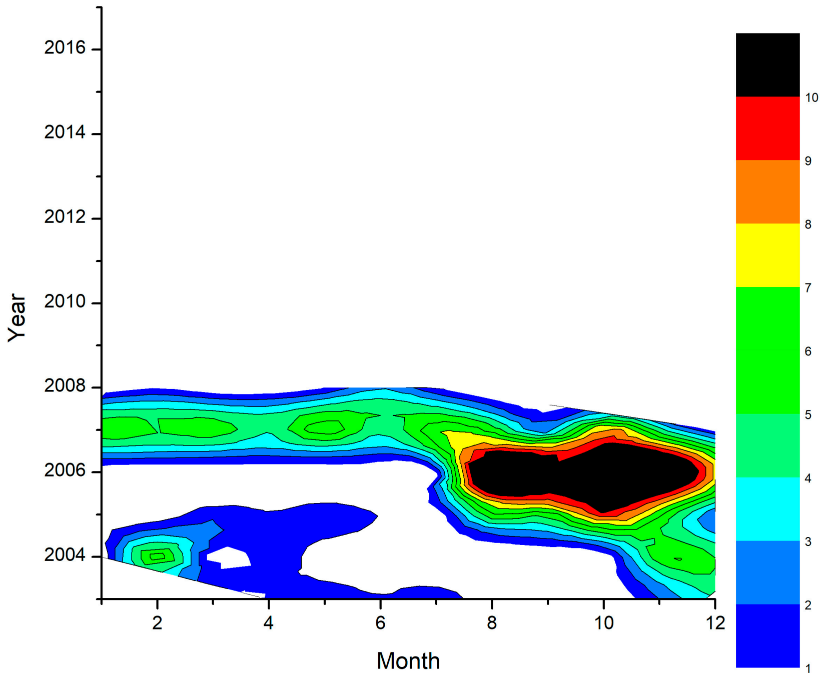

2016
2014
2012
2010
2008
2006
2004
Year
2
4
6
8
10
12
Month
10
9
8
7
6
5
4
3
2
1

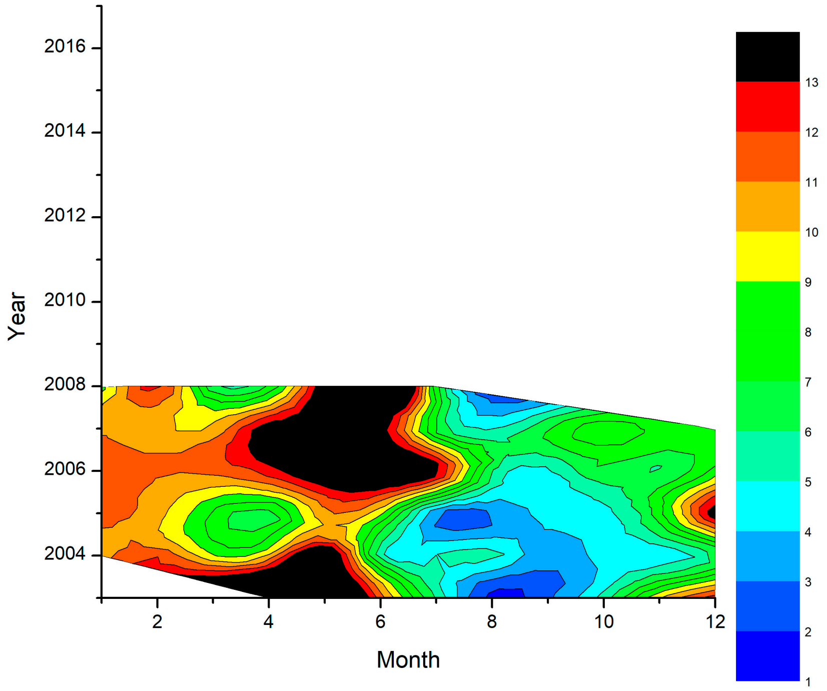

2016
2014
2012
2010
2008
2006
2004
Year
2
4
6
8
10
12
Month
13
12
11
10
9
8
7
6
5
4
3
2
1

(m)

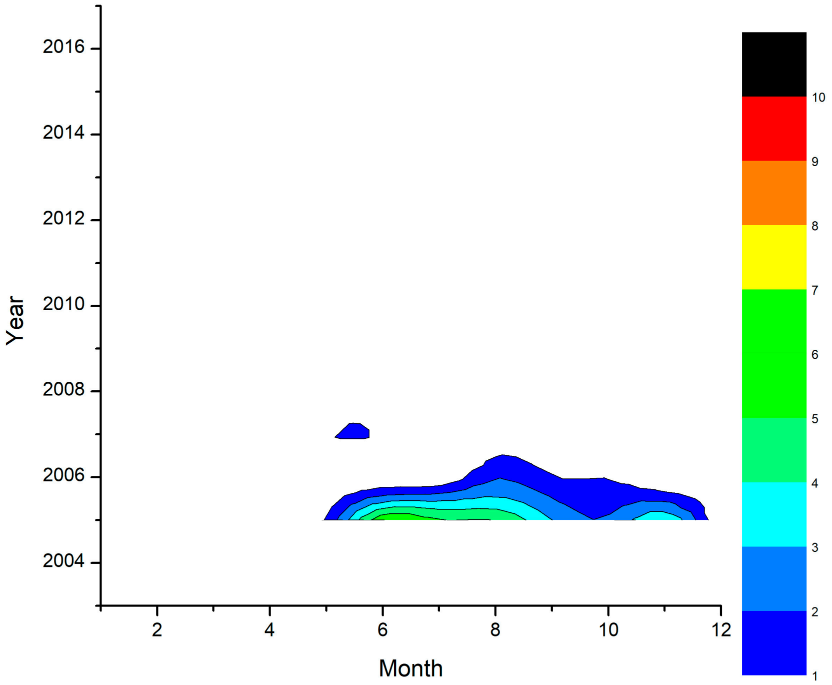

2016
2014
2012
2010
2008
2006
2004
Year
2
4
6
8
10
12
Month
10
9
8
7
6
5
4
3
2
1

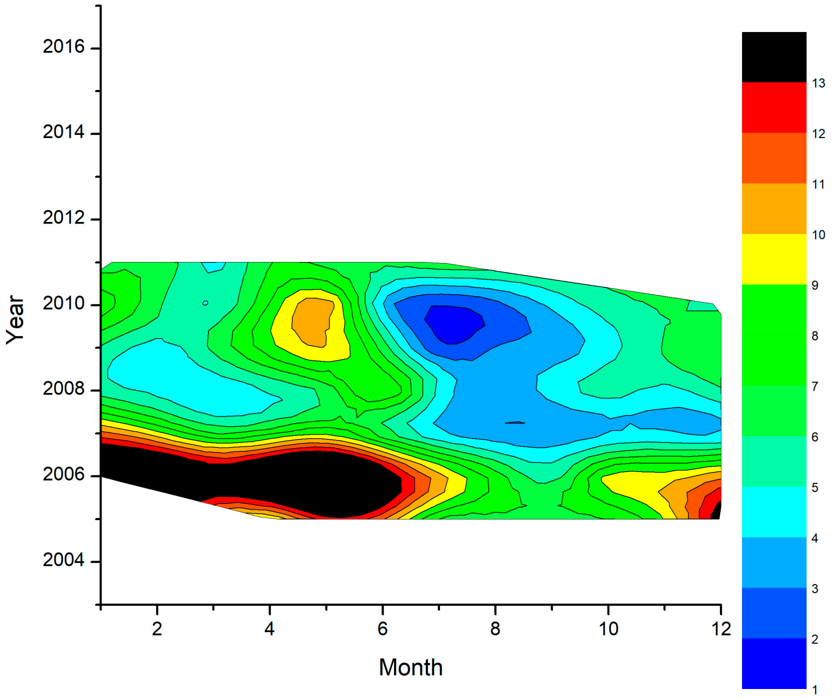

2016
2014
2012
2010
2008
2006
2004
Year
2
4
6
8
10
12
Month
13
12
11
10
9
8
7
6
5
4
3
2
1

(n)

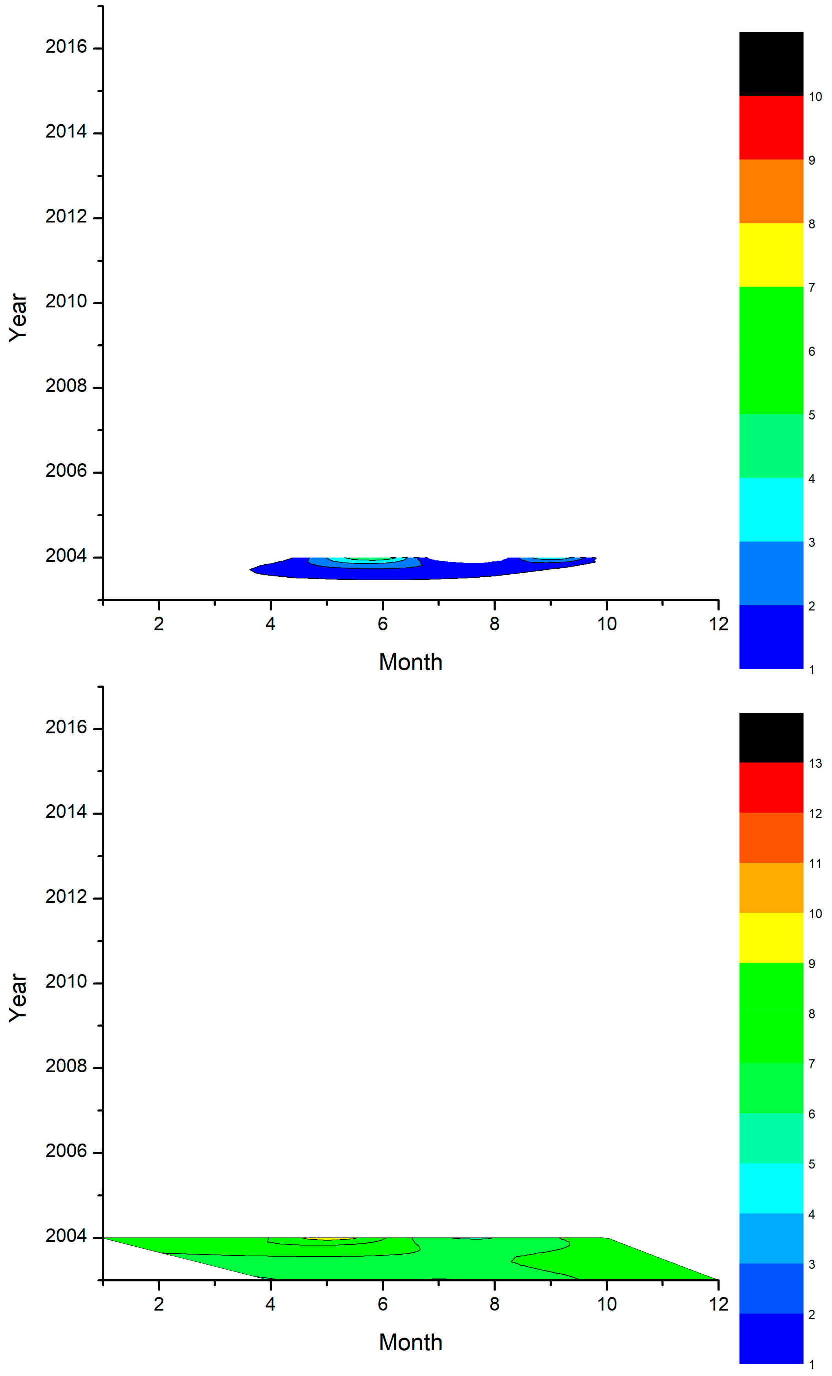

2016
2014
2012
2010
2008
2006
2004
Year
2
4
6
8
10
12
Month
10
9
8
7
6
5
4
3
2
1
2016
2014
2012
2010
2008
2006
2004
Year
2
4
6
8
10
12
Month
13
12
11
10
9
8
7
6
5
4
3
2
1

(o)

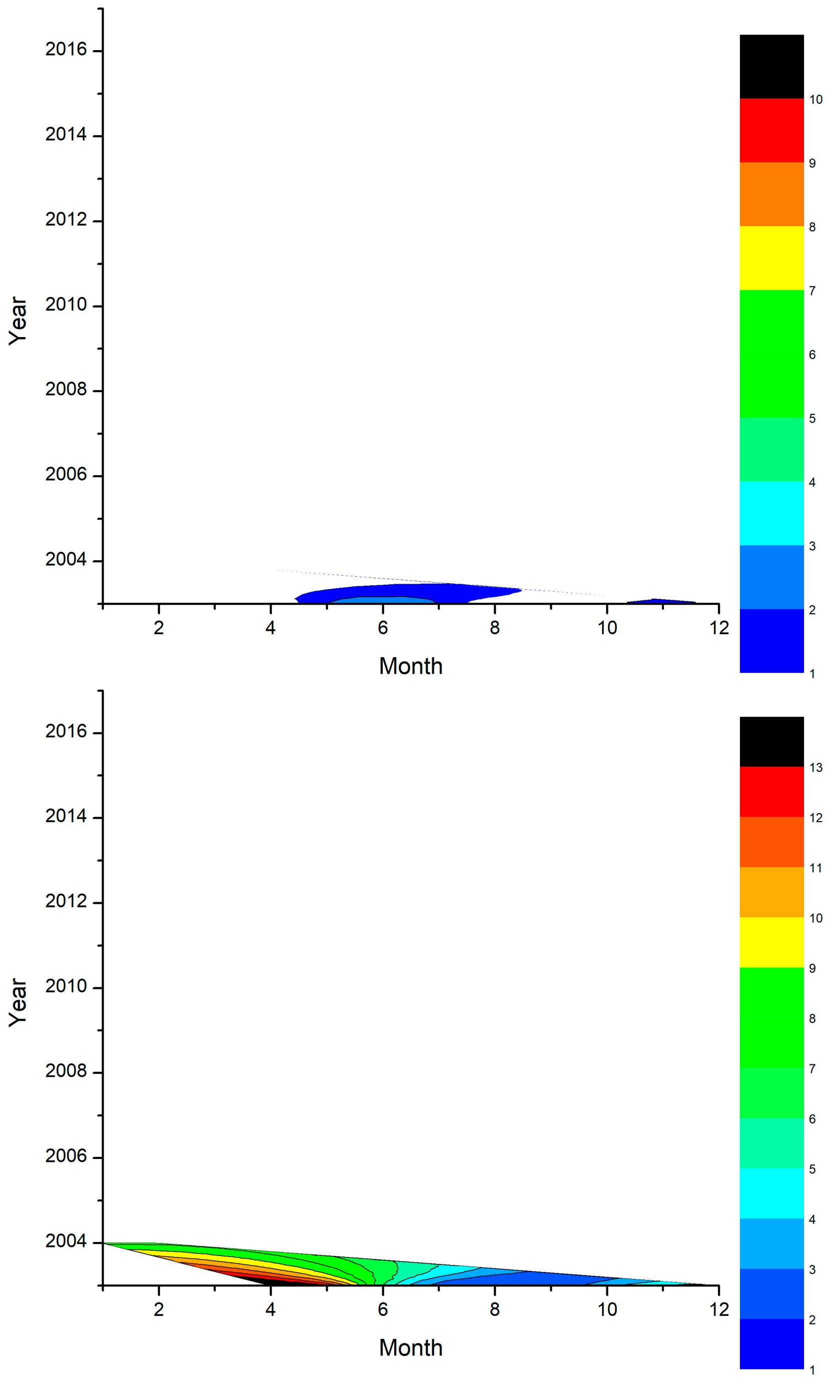

Year
Month
2016
2014
2012
2010
2008
2006
2004
2
4
6
8
10
12
1
2
3
4
5
6
7
8
9
10
11
12
13

(p)

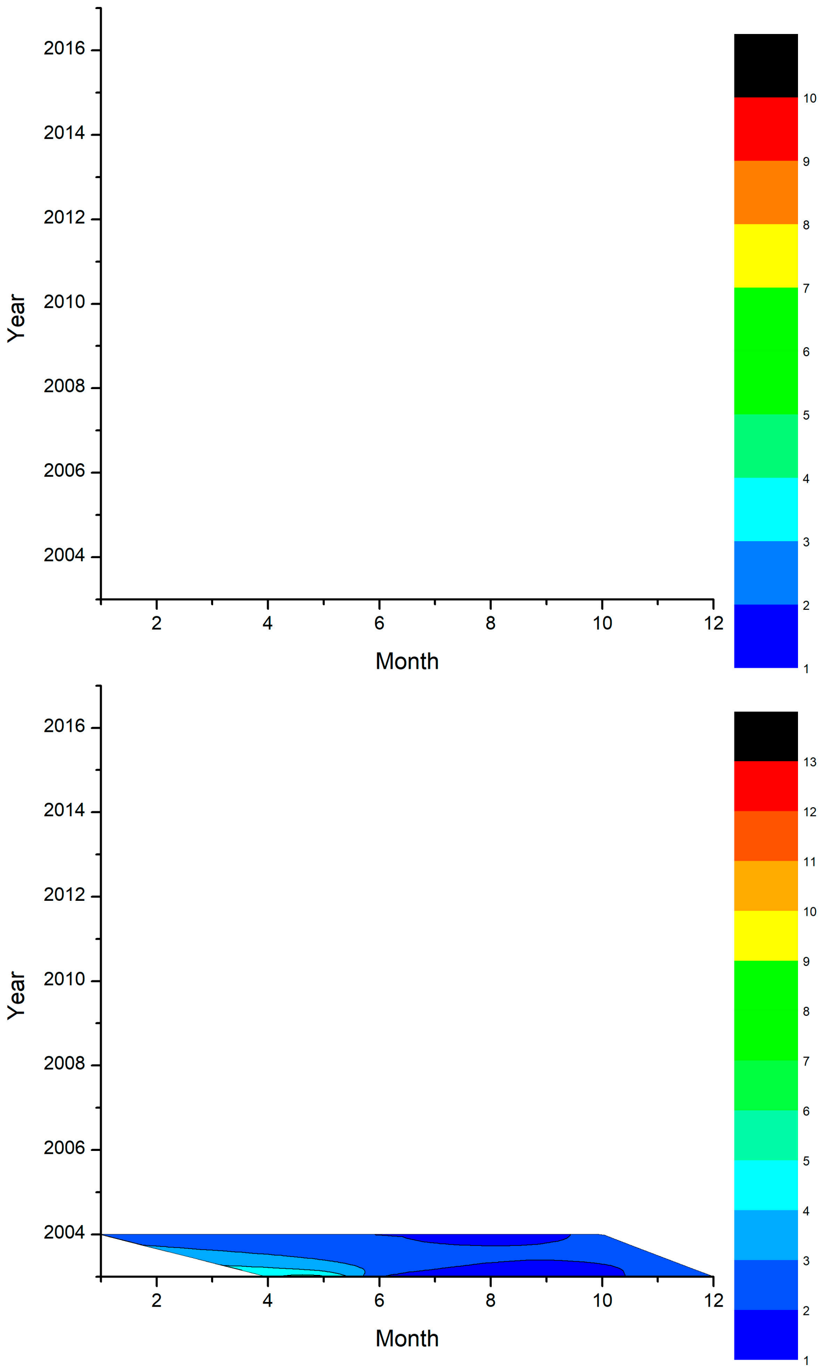
2016
2014
2012
2010
2008
2006
2004
Year
2
4
6
8
10
12
Month
10
9
8
7
6
5
4
3
2
1
2016
2014
2012
2010
2008
2006
2004
Year
2
4
6
8
10
12
Month
13
12
11
10
9
8
7
6
5
4
3
2
1

(q)

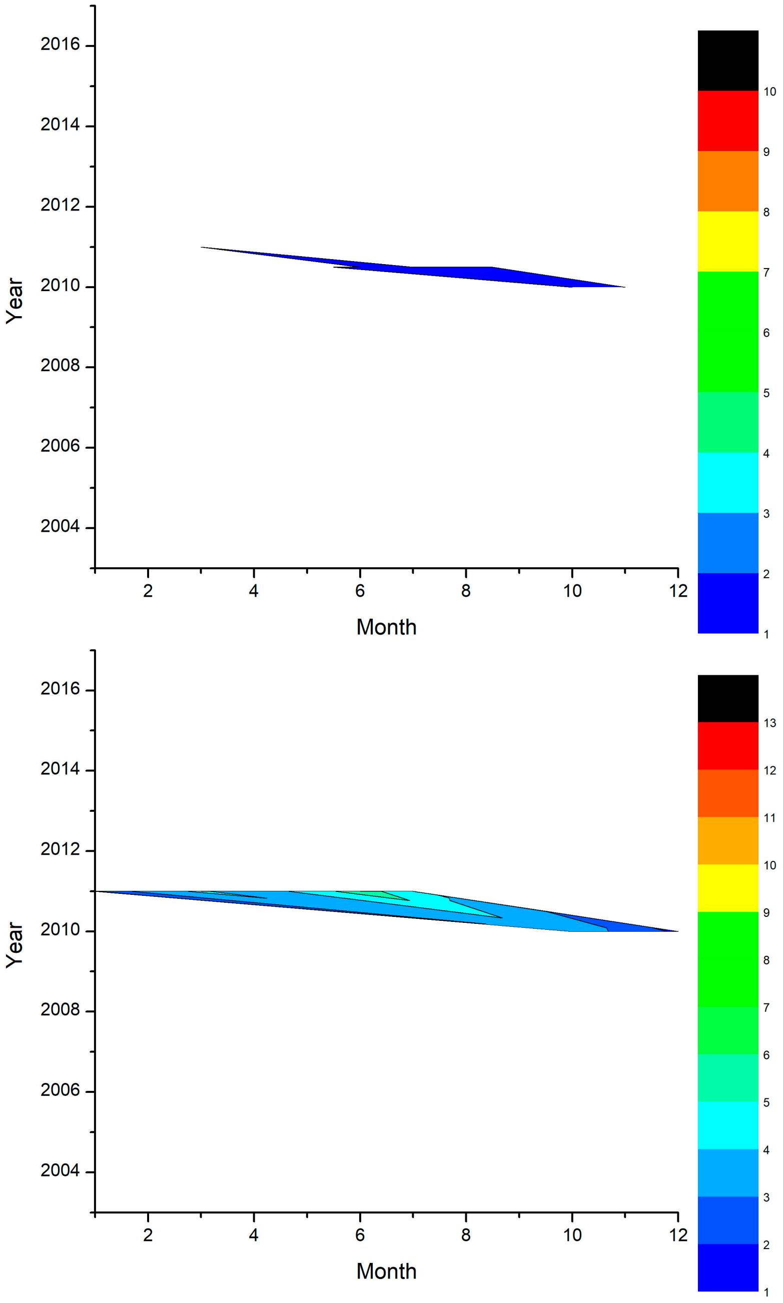

(r)

Figure S1. The contour maps of feces output (kg) and frequency of mucous stools (times) for each panda at Beijing Zoo from 2003 to 2017. The upper panel in each figure indicates the mucoid occurrence and the lower panel indicates the feces output for each panda. (**a**) Benben; (**b**) Jinghui; (**c**) Dabaitu; (**d**) Xiaobaitu; (**e**) Fulu; (**f**) Mengmeng; (**g**) Gugu; (**h**) Dadi; (**i**) Lele; (**j**) Jini; (**k**) Mengda; (**l**) Menger; (**m**) Niuniu; (**n**) Yinghua; (**o**) Yingying; (**p**) Youyou; (**q**) Yuanyuan; (**r**) Wenwen.

Table S1. Giant pandas used in this study.

Studbook#	Name	Year of birth	Sex	Time window of data collection	Translocate history/Death Date	
320	LeLe	1986	F	2003.4–2017.6		
369	YingYing	1991	M	2003.4–2004.10	Death	Dec 13 2006
345	YouYou	1988	M	2003.4–2004.2	Death	Feb 04 2004
					BJ-WL	18 Jun 2006
					WL-BJ	15 Mar 2007
					BJ-YA	27 Jun 2010
					YA-BJ	2 Nov 2010
					BJ-YA	4 Mar 2011
403	JiNi	1993	F	2003.4–2017.6	YA-BJ	24 Aug 2011
					BJ-YA	18 Feb 2012
					YA-BJ	3 Sep 2012
					BJ-YA	21 Feb 2013
					YA-BJ	18 Sep 2013
					BJ-YA	20 Apr 2014
					YA-BJ	5 Sep 1995
421	NiuNiu	1995	F	2003.4–2008.7	BJ-WL	18 Jun 2006
					WL-BJ	9 Jul 2006
					Death	22 Jul 2008
450	BenBen	1997	M	2003.4–2007.11	Death	02 Jan 2008
469	JingHui	1998	M	2003.4–2009.7	Death	03 Nov 2009
488	YuanYuan	1999	M	2003.4–2004.10		
394	DaDi	1992	M	2004.10–2017.6		
496	GuGu	1999	M	2004.11–2017.6		
566	YingHua	2003	F	2005.4–2011.7		
247	WenWen	1982	F	2010.10–2011.7	Death	23 May 2013
652	MengMeng	2006	F	2008.1–2011.7		
784	XiaoBaiTu	2010	F	2012.8–2016.4		
766	DaBaiTu	2010	F	2012.8–2017.2		
883	FuLu	2013	F	2014.11–2017.6		
894	MengDa	2013	M	2014.11–2017.6		
895	MengEr	2013	M	2014.11–2017.6		

BJ: Beijing, WL: Wolong BC, YA: Yaan BC
